# Impact of host factors on susceptibility to antifungal agents

**DOI:** 10.5599/admet.1164

**Published:** 2022-01-07

**Authors:** Balbina Plotkin, Monika Konaklieva

**Affiliations:** 1 Department of Microbiology and Immunology, College of Graduate Studies, Midwestern University, Downers Grove, IL 60515; 2 Department of Chemistry, American University, College of Arts and Sciences, 4400 Massachusetts Ave, NW, Washington, DC 20016

**Keywords:** Insulin, glucose, human serum, *Candida*, *Cryptococcus*

## Abstract

An obstacle to drug development, particularly in this era of multiple drug resistance, is the under-appreciation for the role the host environment plays in microbial response to drugs. With the rise in fungal infection and drug resistance, particularly in individuals with co-morbidities, the influence serum and its components have on antimicrobial susceptibility requires assessment. This study examined the impact of physiologically relevant glucose and insulin levels in the presence and absence of 50 % human plasma on MICs for clinical isolates of *Candida lusitaniae, Candida parapsilosis, Candida albicans, Candida tropicalis, Candida glabrata, Candida krusei* and *Cryptococcus neoformans*. The addition of insulin or glucose at physiologic levels in RPMI medium alone altered the MIC in either a positive or negative fashion, depending on the organisms and drug tested, with *C. glabrata* most significantly altered with a 40, >32- and 46-fold increase in MIC for amphotericin B, itraconazole and miconazole, respectively. The addition of candida-antibody negative plasma also affected MIC, with the addition of glucose and insulin having a tandem effect on MIC. These findings indicate that phenotypic resistance of *Candida* and *Cryptococcus* can vary depending on the presence of insulin with glucose and plasma. This modulation of resistance may help explain treatment failures in the diabetic population and facilitate the development of stable drug-resistant strains. Furthermore, these findings indicate the need for a precision approach in the choice of drug treatment and drug development.

## Introduction

Phenotypic response to environmental shifts is essential for microbial survival and is of particular importance with respect to changes that occur *in vivo*, which affect response to antimicrobial agents. The potential changes in response to drugs upon growth within the host could both be responsible for treatment failures and provide the potential for novel drug development. Thus, it is essential to define these phenotypic responses to host factors, and metabolic conditions, to predict effective drug design and utility.

A common metabolic condition associated with increased risks for infections is type II diabetes. People with type II diabetes can exhibit hyperglycemia, hyperinsulinemia, and/or combined hyperglycemia-hyperinsulinemia, as a result of insulin intolerance [1,2]. A metabolic immunosuppressive condition, type II diabetes, places individuals at increased risk for fungal diseases [3-6]. Thus, during the infectious process, a pathogen can be exposed to elevated levels of insulin and/or glucose as compared to the metabolically normal host.

A critical disconnect regarding predicted sensitivity via *in vitro* testing and efficacy is the lack of congruency with the metabolic disorders most commonly at risk for fungal infections, e.g., type II diabetes, since antimicrobial activity testing is predicated on the host being in a normal metabolic state. Most studies examining serum effects are focused on the impact normal serum/plasma has on antimicrobial activity, or on the effect of high glucose (2 mg/dl) concentrations, which are reported to affect the ability of antifungal agents to bind to their targets [7-9]. To date, studies of glucose-fungal-antifungal agent interactions fail to take into consideration the concomitant presence of insulin with glucose. This becomes important since organisms across the taxonomic kingdoms are reported to produce and/or react to insulin [10-16]. In *Candida albicans*, insulin has been associated with increased expression of virulence-associated morphological transition, from blastospore to hyphal production [17]. The mechanism via which insulin promotes yeast to hyphal transition is by enhancing uptake of proline by *C. albicans*. Conversely, glucose concentrations can affect morphogenic expression [18,19]. In the present study, the effect on *in vitro* antifungal activity in the presence and absence of human plasma by insulin and/or glucose, at levels analogous to those reported in type II diabetes, was determined.

## Experimental

### Fungal species and culture

*Candida lusitaniae, Candida parapsilosis, Candida albicans, Candida tropicalis, Cryptococcus neoformans, Candida glabrata*, and *Candida krusei* clinical isolates (a generous gift from the lab of Paul Schreckenberger, Loyola University Medical School) were maintained at -70 °C and passed at least twice on Sabouraud dextrose agar (SDA) prior to testing.

### Drug testing

Antifungal activity determinations were done using the standard CLSI (NCCLS M27-T) microbroth dilution method with modifications [20,21]. Susceptibility to amphotericin B (Sigma-Aldrich), fluconazole (Pfizer), itraconazole (Janssen Pharmaceuticals), and miconazole (Sigma-Aldrich) was determined using antifungal agents constituted and serially diluted. Microtiter plates containing the serial dilutions of the antifungal agents were made as recommended and stored frozen (-70 °C; ≤ 7 days) or refrigerated (itraconazole; ≤ 3 days). Antifungal activity in the presence or absence of normal human plasma (type A, Rh +), was performed using 2X RPMI medium (50 % v/v). Plasma was screened for specific fungal antibodies, as described below, by indirect immunofluorescent microscopy (FITC-Protein A) to detect the presence of yeast-bound antibodies. Minimum lethal concentrations (MLC) were determined by plating 10 μl of all wells showing no growth onto SDA.

To simulate glucose and insulin levels reported for type II diabetes, glucose (285 mg/dl) and/or insulin (200 μU/ml), final concentrations were tested in RPMI with and without 50 % plasma medium. Endogenous glucose levels were determined using a kit according to the manufacturer’s instructions (Sigma-Aldrich). The reported mid-range value for normal human insulin levels (18 μU/ml) was used as the basis for calculating the amount of insulin to add. Glucose and/or insulin in RPMI medium had no effect (data not shown) on fungal growth kinetics as determined by both turbidimetric measurements (absorbance 560 nm) and a direct microscopic count of yeast and hyphal forms. The growth rates and the relative production of yeast and hyphal forms during growth in RPMI medium with and without glucose and/or insulin were measured to determine if changes in the MIC and MLC of the antifungal agents tested in the presence of glucose and/or insulin could be correlated to alterations in fungal growth. No significant differences in generation time or proportion of yeast and hyphal forms were measured. The differences in the rate of growth between organisms also appear to be unrelated to the alterations in MIC and MLC measured in the presence of glucose and/or insulin and to the relative susceptibility of the organisms. The antifungal agents were not exposed to the media supplements (glucose and insulin) until organisms were added to the agent. Sterility controls and growth controls for all test conditions were run simultaneously with the tests.

To determine antifungal activity in the presence of normal human plasma (type A, Rh +), a modified protocol was developed. Plasma was screened for specific fungal antibodies by indirect immunofluorescent microscopy using FITC-Protein A to detect the presence of bound antibodies. Equal volumes of normal human plasma were added to 2X NCCLS RPMI medium to yield a medium that contained 1X RPMI with 50 % v/v of plasma. For glucose in a medium containing 50 % plasma (plasma medium), glucose and/or insulin were added to ensure equivalent amounts were used for the medium alone. Antifungal agents were prepared, as described above, in this plasma medium. Antifungal activity in the presence and absence of plasma was determined on the same day using the same drug lot and microbial suspension. Minimum inhibitory concentrations (MIC) were determined by visual inspection of the wells at 24, 36 and 72 hrs after initiation of the incubation period. Minimum lethal concentrations (MLC) were determined by plating 10 μl of all wells showing no growth onto Sabouraud dextrose agar (Difco) at 36 hrs post-initiation of the incubation period.

## Results and discussion

### Amphotericin B

The overall effect of insulin and glucose alone or together were drug and fungal species-specific (Table 1). Amphotericin B (AmB) activity was relatively unaffected by insulin and glucose combined with the exception of *C. glabrata* (40-fold greater MIC than RPMI alone; Table 1). For AmB, the presence of plasma with and without glucose and/or insulin decreased the MIC < 4 fold of that measured for medium alone for all organisms, with the exception of *C. glabrata* and *C. neoformans*. For amphotericin B in plasma supplemented with glucose, its MIC increased from four to a maximum of 32-fold for *C. krusei* compared to medium with plasma alone (Table 2). Like with itraconazole, with amphotericin B, there was a difference between the MIC and MLC. The MLC of *C. albicans* was 32-fold higher than the MIC in the presence of plasma without supplements. The plasma effect on the candidal MLC was overcome by the addition of glucose and/or insulin. The MLC was within 2-fold of the amphotericin B MIC for all other isolates. Comparison of medium with plasma to medium alone (Table 3) indicated that the addition of 50 % normal human plasma to medium decreased the MIC of amphotericin B > 16-fold for all isolates, except *C. lusitaniae* and *C. krusei*, which were unaffected.

### 5-Fluorocytosine

The MIC of 5-fluorocytosine in media supplemented with glucose alone either was unaffected *(C. lusitaniae, C. neoformans*, and *C. krusei*), or decreased (Table 1). The effect of insulin on the MIC in media was also isolate-dependent. Insulin alone had the greatest negative impact on 5-fluorocytosine activity for *C. tropicalis* (~25-fold), with *C. parasilosis* and *C. glabrata* exhibiting a ~6-fold increase in MIC. In general, the MIC of all isolates in medium supplemented with glucose and insulin was decreased compared to medium alone, although the degree of decrease was isolate-dependent. The presence of plasma (50 % v/v RPMI medium) resulted in an increase in 5-fluorocytosine MIC maximally for *C. parasilosis*, (32-fold) and *C. tropicalis* (16-fold), as compared to RPMI medium alone for all isolates, with the exception of *C. glabrata*, whose MIC was markedly decreased (Table 2). The MLC in media, and in the presence of plasma with or without supplements correlated within two-fold of the MIC, with the exception of *C. glabrata*, which in the presence of plasma was four-fold higher than that measured for media alone. The addition of insulin to both medium alone, and with plasma, resulted in an increase in the MIC to a maximum increase measured for *C. glabrata* when exposed to 5-fluorocytosine (40-fold increase; 25 μg/ml in media with plasma and insulin vs. 0.625 μg/ml media with insulin alone). Comparison of medium with plasma, to medium alone (Table 3), indicated that the addition of 50 % normal human plasma to medium decreased the MIC 25-fold for *C. tropicalis* only (0.25 μg/ml media with 50 % plasma vs. 6.25 μg/ml RPMI medium alone).

### Fluconazole

Supplementation of RPMI medium containing plasma with insulin, resulted in a 1024-fold increase in MIC of *C. albicans* to fluconazole with the resultant reclassification from sensitive to resistant. However, addition of glucose to insulin restored the sensitivity of *C. albicans* to fluconazole. The only change in classification with regards to fluconazole was a shift from sensitive to sensitive-dose dependent for *C. lusitaniae* when both glucose and insulin were present. In contrast, the presence of plasma resulted in a shift from resistant to sensitive for *C. neoformans* with respect to fluconazole.

### Itraconazole

For itraconazole, the addition of glucose and/or insulin to RPMI medium did not significantly alter the MIC or MLC (≤ 2-fold of MIC in medium alone) for all organisms with the exception of *C. lusitaniae* and *C. glabrata* (Table 1). The addition of glucose and/or insulin to RPMI medium resulted in a MIC for *C. glabrata* that exceeded the drug solubility level (see Table 1). The effect of glucose on the *C. lusitaniae* response to itraconazole was opposite of its effect when combined with insulin (8-fold higher MIC for glucose alone vs. 8-fold lower MIC for glucose and insulin), indicating that there may be an interactive effect of glucose and insulin. The presence of plasma alone had a modest (< 4-fold) effect on the MIC for all isolates with the exception of *C. glabrata*, where the MIC was decreased nearly 103-fold as compared to that measured for medium alone (Table 2 and 3). The addition of glucose and/or insulin to plasma did not significantly alter the MIC or MLC of itraconazole for all organisms, except for *C. neoformans*, where supplementing plasma with insulin resulted in a 20-fold decrease in the MIC. Interestingly, the MLC for all conditions tested differed ≤ 2-fold from the MIC for all organisms, except for *C. neoformans* (8-fold higher than MIC in plasma supplemented with glucose and insulin) and C*. glabrata* (128-fold higher than MIC in plasma supplemented with glucose). The MLC for *C. neoformans* in RPMI alone was less affected, exhibiting a five-fold decrease in MLC compared to an MLC measured in plasma alone, or plasma supplemented with glucose. This differential effect of the presence of plasma, as compared to medium alone, may explain the treatment failure reported despite laboratory-confirmed sensitivity [22-27].

### Miconazole

The combination of glucose and insulin resulted in a significant alteration in the miconazole MIC for all isolates except *C. parapsilosis*. Resistance to miconazole was increased by the presence of insulin and glucose to a maximum of 46-fold and 23-fold over that of RPMI alone for *C. glabrata* and *C. krusei*, respectively (Table 1). The MIC in media supplemented with glucose was decreased from four-fold to a maximum 16-fold measured for *C. albicans* in miconazole. *C. neoformans* was the only organism whose MIC was affected by the presence of insulin. Interestingly, while plasma alone significantly increased drug activity for all drugs tested against *C. glabrata*, the addition of insulin and/or glucose restored this activity to the levels measured for RPMI with plasma alone (Tables 2 and 3). In contrast, the presence of plasma negatively impacted miconazole (*C. lusitaniae*, > 185-fold; *C. tropicalis*, > 23-fold; *C. krusei*, > 92-fold) and with the addition of glucose and insulin, MIC returned to levels within 4-fold that of RPMI with plasma. The addition of glucose to plasma caused either a decrease in the MIC or did not alter the MIC. In all test situations, the MLC correlated with the MIC.

Thus, the addition of plasma to the medium resulted in an overall decrease in the concentration of miconazole, itraconazole, 5-fluorocytosine, and amphotericin B needed to inhibit the growth of *C. glabrata* (Table 3). In contrast, plasma overall caused an increase in the concentration of miconazole, itraconazole, 5-fluorocytosine, and amphotericin B needed to inhibit the growth of *C. lusitaniae, C. tropicalis* and *C. krusei*. The presence of the medium supplements insulin and/or glucose does not affect fungal MIC or MLC in a predictable manner regarding specific antifungal agents or organisms tested. The growth rate and relative production of yeast and/or hyphal forms in medium with and without glucose and/or insulin do not appear to be related to changes in the organism's MIC or MLC.

## Conclusions

The testing of fungal susceptibility in the presence of plasma with and without the supplements glucose and/or insulin to simulate type II diabetes appears warranted in light of their sometimes adverse effect on the MIC of the antifungal agents. These results taken together indicate that alterations in the levels of serum components associated with type II diabetes, and simulated herein, cause an alteration in the *in vitro* activity of certain antifungal agents that is also dependent on the presence of plasma. Since treatment failure in candidal infections has been an issue and is increasing, it is likely that a portion of treatment efficacy failure is due to phenotypic resistance [28,29]. While multiple *in vitro* studies show that glucose induces phenotypic resistance, the organism is always exposed to glucose in the presence of insulin *in vivo* [27,30,31]. Most commonly, these studies focused on the effect glucose has on end-point determinations for amphotericin B, fluconazole and itraconazole [32]. This study demonstrated that testing of individual serum components, e.g., insulin and glucose *in vitro* showed that not only does insulin or glucose affect susceptibility in a manner that was species and drug-specific, but that the interaction of the two components can negate the effect, either positive or negative, of the other additive. This impact of insulin and glucose is further compounded by testing in the presence of human plasma vs. RPMI medium alone. These unpredictable phenotypic changes in susceptibility to antifungal agents were demonstrated for amphotericin B and *C. glabrata*, which were sensitive to the drug in medium alone or supplemented with either glucose or insulin. However, when combined, the MIC was > 40-fold higher than medium alone or with either glucose or insulin. This effect was eliminated with the addition of anti-candidal antibody-negative plasma. In summary, these findings present evidence that a precision approach is needed for the determination of fungal drug susceptibility, particularly for individuals with type II diabetes.

## Figures and Tables

**Table 1.** Effect of insulin and glucose on fungal MIC (μg/ml)

Organism	Drug	RPMI	RPMI + Gluc^c^	Ratio RPMI + Gluc RPMI	RPMI + Insu^d^	Ratio RPMI + Insu RPMI	RPMI + Glucose & Insulin	Ratio RPMI + Gluc & Insu RPMI
*C. lusitaniae*	AmB^a^	0.039 (S) ^b^	0.0195 (S)	0.5	0.0195 (S)	0.5	0.078 (S)	2
	5-FC^a^	0.15625 (S)	0.15625 (S)	1	0.15625 (S)	1	0.00936 (S)	0.0599
	Flucon^a^	4.0 (S)	8.0 (S)	2	2.0 (S)	0.5	16.0 (S-DD)	4
	Itra^a^	0.5 (S-DD)^b^	4.0 (R) ^b^	8	0.5 (S-DD)	1	0.0625 (S)	0.125
	Micon^a^	0.195 (S)	0.195 (S)	1	0.195 (S)	1	2.2425 (S)	11.5
*C. parapsilosis*	AmB	0.078 (S)	0.0195 (S)	0.25	0.039 (S)	0.5	0.078 (S)	1
	5-FC	0.78 (S)	0.39 (S)	0.5	4.99 (I) ^b^	6.4	0.312 (S)	0.4
	Flucon	2.0 (S)	8.0 (S)	4	2.0 (S)	1	8.0 (S)	4
	Itra	0.25 (S-DD)	0.25 (S-DD)	1	0.25 (S-DD)	1	0.25 (S-DD)	1
	Micon	0.39 (S)	0.78 (S)	2	0.39 (S)	1	0.546 (S)	0.71
*C. albicans*	AmB	0.039 (S)	0.0195 (S)	0.5	0.0195 (S)	0.5	0.039 (S)	1
	5-FC	0.78 (S)	0.1 (S)	0.1299	0.39 (S)	0.5	0.078 (S)	0.1
	Flucon	0.125 (S)	0.125 (S)	1	0.125 (S)	1	0.5 (S)	4
	Itra	0.03125 (S)	0.0156 (S)	(2)	0.03125 (S)	1	0.0625 (S)	2
	Micon	0.39 (S)	0.0245 (S)	0.0629	0.78 (S)	2	0.01757 (S)	0.045
*C. tropicalis*	AmB	0.039 (S)	0.0195 (S)	0.5	0.039 (S)	1	0.156 (S)	4
	5-FC	0.195 (S)	0.0975 (S)	0.5	4.99 (I)	25.6	0.156 (S)	0.8
	Flucon	>128.0 (R)	>128.0 (R)	≥1	>128 (R)	>1	>128 (R)	≥1
	Itra	>64.0 (R)	>64.0 (R)	≥1	>64 (R)	≥1	>64 (R)	≥1
	Micon	1.56 (S)	0.78 (S)	0.5	1.56 (S)	1	9.048 (R)	5.8
*C. neoformans*	AmB	0.039 (S)	0.0195 (S)	0.5	0.039 (S)	1	0.039 (S)	1
	5-FC	6.25 (I)^b^	6.25 (I)	1	0.78 (S)	0.125	0.78 (S)	0.125
	Flucon	8.0 (R)	8.0 (R)	1	8.0 (R)	1	8.0 (R)	1
	Itra	0.5 (S-DD)	0.5 (S-DD)	1	0.5 (S-DD)	1	0.5 (S-DD)	1
	Micon	0.9766 (S)	0.1953 (S)	0.2	0.09766 (S)	0.1	4.492 (S)	4.6
*C. glabrata*	AmB	0.078 (S)	0.0195 (S)	0.25	0.039 (S)	0.5	3.12 (R)	40
	5-FC	8.0 (I)	4.0 (I)	0.5	51.2 (R)	6.4	1.6 (I)	0.2
	Flucon	0.09766 (S)	0.3906 (S)	4	0.195 (S)	2	0.195 (S)	2
	Itra	2.0 (R)	>64 (R)	>32	>64 (R)	>32	>64 (R)	>32
	Micon	0.39 (S)	0.78 (S)	2	0.39	1	17.94 (R)	46
*C. krusei*	AmB	0.078 (S)	0.039 (S)	0.5	0.078 (S)	1	0.078 (S)	1
	5-FC	12.5 (I)	12.5 (I)	1	12.5 (I)	1	5.0 (I)	0.4
	Flucon	64.0 (R)	64.0 (R)	1	64.0 (R)	1	64.0 (R)	1
	Itra	1.0 (R)	1.0 (R)	1	1.0 (R)	1	1.0 (R)	1
	Micon	0.39 (S)	0.78 (S)	2	1.56 (S)	4	8.97 (R)	23

^a^amphotericin B (AmB), flucytosine (5-FC) fluconazole (Flucon), itraconazole (Itra), and miconazole (Micon)

^b^S=sensitive; R=resistant; I=intermediate resistance; S-DD= sensitive based on reported disc diffusion concentration

^c^Gluc=glucose

^d^Insu=insulin

**Table 2.** Effect of insulin and glucose in RPMI medium with human plasma (50 % v/v; RPMI-P) on the MIC (μg/ml)

Organism	Drug	RPMI-P^c^	Ratio RPMI-P RPMI	RPMI-P & Glu^d^	Ratio RPMI-P & Gluc RPMI-P	RPMI-P & Insu^e^	Ratio RPMI-P & Insu RPMI-P	RPMI-P, Gluc & Insu	Ratio RPMI-P, Gluc & Insu RPMI-P
*C. lusitaniae*	AmB^a^	0.078 (S)^b^	2	9.75x10^-3^ (S)	0.25	0.0195 (S)	0.25	0.0195 (S)	0.25
	5-FC^a^	0.78 (S)	5	0.78 (S)	1	0.78 (S)	1	0.78 (S)	1
	Flucon^a^	1.0 (S)	0.25	1.0 (S)	1	4.0 (S)	4	4.0 (S)	1
	Itra^a^	2.0 (R) ^b^	4	2.0 (R)	1	2.0 (R)	1	2.0 (R)	1
	Micon^a^	>36.075 (R)	>185	>36.075 (R)	≥1	0.3608 (S)	0.01	>36.08 (R)	≥1
*C. parapsilosis*	AmB	0.0195 (S)	0.25	0.078 (S)	4	0.039 (S)	2	0.0195 (S)	1
	5-FC	24.96 (I) ^b^	32	24.96 (I)	1	24.96 (I)	1	24.96 (I)	1
	Flucon	1.0 (S)	0.5	0.5 (S)	0.5	8.0 (S)	8	0.5 (S)	0.5
	Itra	0.125 (S)	0.5	0.125 (S)	1	0.125 (S)	1	0.5 (S-DD)	2
	Micon	>39.88 (R)	>92	>39.88 (R)	1	>39.88 (R)	≥1	>39.88 (R)	≥1
*C. albicans*	AmB	0.00975 (S)	0.25	0.0195 (S)	2	0.0195 (S)	2	0.0195 (S)	2
	5-FC	3.12 (S)	4	3.12 (S)	1	3.12 (S)	1	3.12 (S)	1
	Flucon	0.075 (S)	0.5	0.075 (S)	1	>6.8 (S-R)	1024	0.3 (S)	4
	Itra	0.03125 (S)	1	0.03125 (S)	1	0.03125 (S)	1	0.03125 (S)	1
	Micon	0.39 (S)	1	0.065 (S)	0.166	0.0325 (S)	0.083	0.78 (S)	2
*C. tropicalis*	AmB	0.0195 (S)	0.5	0.0195 (S)	1	0.0195 (S)	1	0.0195 (S)	1
	5-FC	3.12 (S)	16	3.12 (S)	1	3.12 (S)	1	3.12 (S)	1
	Flucon	>128 (R)	>1	>128 (R)	1	>128.0 (R)	>1	>128.0 (R)	1
	Itra	>64 (R)	1	>64 (R)	1	>64.0 (R)	1	>64.0 (R)	1
	Micon	>35.88 (R)	>23	>35.88 (R)	1	>35.88 (R)	≥1	>35.88 (R)	≥1
*C. neoformans*	AmB	0.00488 (S)	0.125	0.00975 (S)	2	0.00975 (S)	1	0.00975 (S)	2
	5-FC	25.0 (I-R)	4	12.5 (I)	0.5	12.5 (I)	0.5	12.5 (I)	0.5
	Flucon	1.0 (S)	0.125	0.25 (S)	0.25	1.0 (S)	1	2.0 (S)	2
	Itra	0.25 (S-DD)^b^	0.5	0.25 (S-DD)	1	0.0125 (S)	0.05	0.25 (S-DD)	1
	Micon	1.953 (S)	2	0.3906 (S)	0.2	0.078 (S)	0.04	7.812 (R)	4
*C. glabrata*	AmB	3.71x10^-5^ (S)	4.76x10^-4^	3.7 x 10^-5^ (S)	1	3.71x10^-5^(S)	1	3.71x10^-5^ (S)	1
	5-FC	0.0615 (S)	7.69x10^-3^	<0.0615 (S)	<1	<0.0615 (S)	≤1	0.246 (S)	4
	Flucon	<2.4x10^-5^(S)	<2.4x10^-4^	<2.4x10^-5^ (S)	<1	<2.4x10^-5^(S)	1	<2.4x10^-5^(S)	≤1
	Itra	0.0194 (S)	9.7x10^-3^	0.0194 (S)	1	0.0194 (S)	1	0.0194 (S)	1
	Micon	1.08x10^-3^ (S)	2.8x10^-3^	5.41 x 10^-4^(S)	0.5	0.0964 (S)	89	0.001083 (S)	1
*C. krusei*	AmB	0.156 (S)	2	0.624 (S)	4	0.156 (S)	1	0.156 (S)	1
	5-FC	>25.0 (I-R)	>2	>25.0 (I-R)	1	>25.0 (I-R)	≥1	>25.0 (I-R)	≥1
	Flucon	64.0 (R)	1	64.0 (R)	1	64.0 (R)	1	64.0 (R)	1
	Itra	4.0 (R)	4	4.0 (R)	1	4.0 (R)	1	4.0 (R)	1
	Micon	35.88 (R)	>92	8.97 (R)	0.25	574.08 (R)	16	>35.88 (R)	≥1

^a^amphotericin B (AmB), flucytosine (5-FC) fluconazole (Flucon), itraconazole (Itra), and miconazole (Micon)

^b^S=sensitive; R=resistant; I=intermediate resistance; S-DD= sensitive based on reported disc diffusion concentration

^c^P=plasma

^d^Gluc=glucose

^e^Insu=insulin

**Table 3.** Ratio of effects of insulin and glucose on antifungal activity of amphotericin B, flucytosine, fluconazole, itraconazole, and miconazole in the presence of RPMI with 50 % human plasma to homologous RPMI medium alone

Organism	Drug	Ratio RPMI-Plasma RPMI	Ratio RPMI-P^c^ & Gluc^d^ RPMI-Gluc	Ratio RPMI-P & Insu^e^ RPMI-Insu	Ratio RPMI-P, Gluc & Insu RPMI-Gluc & Insu
*C. lusitaniae*	AmB	2.0	0.5	1.0	0.25
	5-FC	5.0	5.0	5.0	83.3
	Flucon	0.25	0.125	2.0	0.25
	Itra	4.0	0.5	4.0	32.0
	Micon	>185.0	>185.0	1.85	16.1
*C. parapsilosis*	AmB	0.25	4.0	1.0	0.25
	5-FC	32.0	64.0	5.0	80.0
	Flucon	0.5	0.0625	4.0	0.0625
	Itra	0.5	0.48	0.5	2.0
	Micon	>92.0	>51.13	>102.3	>73
*C. albicans*	AmB	0.25	1.0	1.0	0.5
	5-FC	4.0	31.2	8.0	40
	Flucon	0.5	0.6	54.4	0.6
	Itra	1.0	2.0	1.0	0.5
	Micon	1.0	2.65	0.042	44.4
*C. tropicalis*	AmB	0.5	1.0	0.5	0.125
	5-FC	16.0	32.0	0.625	20.0
	Flucon	>1.0	1.0	1.0	1.0
	Itra	1.0	1.0	1.0	1.0
	Micon	>23.0	46.0	23.0	3.97
*C. neoformans*	AmB	0.125	0.5	0.25	0.25
	5-FC	4.0	2.0	16.0	16.0
	Flucon	0.125	0.03	0.125	0.25
	Itra	0.5	0.5	0.025	0.5
	Micon	2.0	2.0	0.7987	1.74
*C. glabrata*	AmB	4.76x10^-4^	1.897x10^-3^	9.5x10^--4^	1.2x10^-5^
	5-FC	7.69x10^-3^	1.5x10^-2^	1.2x10^-3^	1.54x10^-1^
	Flucon	<2.4x10^-4^	6.1x10^-5^	1.23x10-4	1.23x10^-4^
	Itra	9.7x10^-3^	3x10^-4^	3x10^-4^	3x10^-4^
	Micon	2.8x10^-3^	6.9x10^-4^	2.47x10^-1^	6.0x10^-5^
*C. krusei*	AmB	2.0	16.0	2.0	2.0
	5-FC	>2.0	2.0	2.0	5.0
	Flucon	1.0	1.0	1.0	1.0
	Itra	4.0	4.0	4.0	4.0
	Micon	>92.0	11.5	368.0	4.0

^a^amphotericin B (AmB), flucytosine (5-FC) fluconazole (Flucon), itraconazole (Itra), and miconazole (Micon)

^b^S=sensitive; R=resistant; I=intermediate resistance; S-DD= sensitive based on reported disc diffusion concentration

^c^P=plasma

^d^Gluc=glucose

^e^Insu=insulin
